# Prevalence of recurrent oral ulcers and association with ABO/Rh group systems in a Lebanese sample^☆^

**DOI:** 10.1016/j.abd.2020.08.039

**Published:** 2022-03-07

**Authors:** Ziad Noujeim, Lara Nasr, Racha Hajj, Abbass El-Outa

**Affiliations:** a Department of Oral and Maxillofacial Surgery, Department of Oral Medicine, Faculty of Dental Medicine, Lebanese University, Beirut, Lebanon; b Attending Oral Surgeon, Baabda University Hospital, Baabda, Lebanon; c Department of Prosthodontics and Esthetic Dentistry, Faculty of Dental Medicine, Saint-Joseph University, Beirut, Lebanon; d Department of Emergency Medicine, American University of Beirut, Beirut, Lebanon

Dear Editor,

Recurrent oral ulcers present a group of diseases characterized by repeated episodes of benign, contagious, or non-contagious and spontaneously-healing ulcerations in otherwise healthy individuals.^1^ These can range from traumatic, infective, aphthous, ulceration related to oral dermatoses, drug-induced, and ulceration as a manifestation of systemic disease.^2^ It is important that an experienced clinician examines oral ulcers and performs the necessary investigations with individuals suffering from recurrent lesions.^3^

Usually, diagnosis is mainly based on the patient’s clinical history.^4^ In most cases, ulcers appear 3 to 6 times per year and last 7 to 10 days.^3^ However, the most common recurrent ulcerative condition of the oral cavity is by far recurrent aphthous stomatitis (RAS).^5^

Several authors have studied the correlation between blood group antigens of recurrent oral ulcers, mainly RAS.^1^, ^3^, ^4^, ^6^^,^^7^ However, to our knowledge, there are no studies on recurrent oral ulcers in the Lebanese population to date. Consequently, this study aims to assess the possible correlation between the ABO blood group and such lesions. Additionally, we report on the prevalence and distribution of recurrent oral ulcers in a sample of the general Lebanese population.

This was a cross-sectional survey study conducted between December 2019 till March 2020 in Lebanon. The survey questionnaire was constructed by a panel of oral medicine professionals. The questionnaire consisted of demographic questions in addition to 15, predominantly close-ended, questions on the participant’s history of oral ulcerations, frequency, period, blood group, and other associated factors. This study was reviewed and approved by the ethical committee of Baabda Governmental University Hospital.

The survey was conducted online and distributed via a participation link that points to the survey. Participants had the option to choose between English and Arabic. The survey was totally voluntary and anonymous. The only inclusion criterion was age older than or equal to 18 years old.

Data was then transferred into and analyzed using IBM SPSS Statistics for Windows, version 24.0 (IBM Corp., Armonk, N.Y., USA). Descriptive statistics were reported for all variables. The Chi-Square tests and Fischer’s exact test were used to assess statistically significant differences between categorical variables. Significance was interpreted at p < 0.05.

We received 249 completed surveys. (125) 50.2% of the respondents were females and (113) 45.4% were males, with (11) 4.4% missing. Age ranged from 18 to 64 years old with a mean of 28.5 ± 9.8 years. Out of the 249 surveyed, 72 (28.9%) answered that they do suffer from recurrent oral ulcers. Females affected were slightly more than males (59.7%), with the age group 18‒25 most affected. Results are presented in Table 1.

**Table 1:** Summary of reported results.

		Frequency	Percent	p
**Gender**	Female	43	59.7	0.126
	Male	29	40.3	
**Age group**	18–24 years	41	56.9	
	25–34 years	21	29.2	
	35–44 years	7	9.7	
	45–54 years	1	1.4	
	55–64 years	2	2.8	
**ABO blood group**	A	32	44.4	
	B	10	13.9	
	O	26	36.1	
	AB	3	4.2	
	No answer	1	1.4	
**Rhesus blood group**	Rh-	6	8.3	
	Rh+	65	90.3	
	No answer	1	1.4	
**ABO Rh**	A-	2	2.8	
	A+	30	41.7	
	AB+	3	4.2	
	B-	1	1.4	
	B+	9	12.5	
	O-	3	4.2	
	O+	23	31.9	
	No answer	1	1.4	
**Number of episodes last year**	1	2	2.8	
	2–4	43	59.7	
	5 or more	26	36.1	
	No answer	1	1.4	
**Site**	Cheek (buccal mucosa)	1	1.4	
	Tongue	4	5.6	
	Lip – labial mucosa	8	11.1	
	Lip – vermillion	4	5.6	
	Gingiva	1	1.4	
	Multiple sites	54	75.0	
**Multiple concomitant ulcers**	Yes	30	41.7	
	No	42	58.3	
**Ulcers correlate with periods of stress**	Maybe	23	31.9	
	No	14	19.4	
	Yes	35	48.6	
**Related to menstrual cycle**	Maybe	8	11.1	
	No	30	41.7	
	Yes	5	6.9	
**Nutritional deficiency (Vit B_12_, iron)**	Maybe	14	19.4	
	No	34	47.2	
	Yes	23	31.9	
	Don’t know	1	1.4	
**Family history of recurrent oral ulcers?**	Maybe	11	15.3	
	No	26	36.1	
	Yes	34	47.2	
	Total	71	98.6	
	Don’t know	1	1.4	
**Daily frequency of toothbrushing**	0	7	9.7	
	1	7	9.7	
	2	37	51.4	
	3	7	9.7	
	No answer	14	19.4	
**Total**		72	100.0	

Mann-Whitney test revealed that young individuals of 18‒24 years old suffered significantly more than other ages (p = 0.001), while older participants had less incidence of recurrent oral ulcers.

The Chi-square test revealed no association between ABO/Rh blood group systems and the presence of recurrent oral ulcers (p > 0.05) (Fig. 1). Blood groups most prevalent in the Lebanese population are A+, and O+ (Table 2), yet groups most commonly affected by recurrent ulcers were A+ (41.7%) and O- (31.9%).

**Figure 1:**
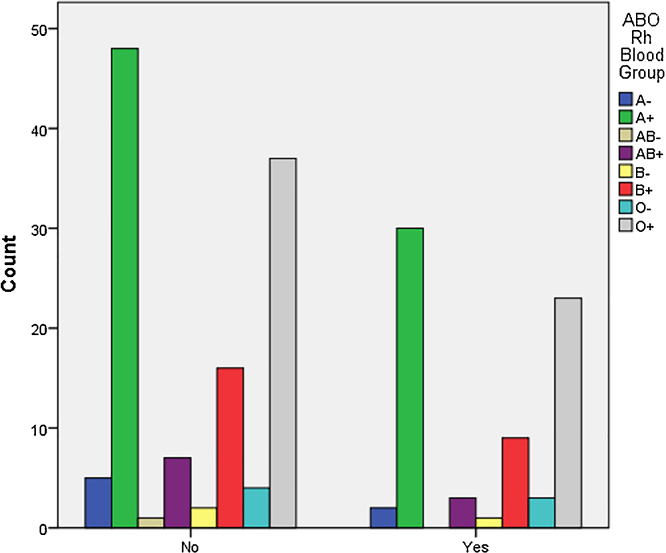
Comparison between blood groups of participants who suffer (Yes) from recurrent aphthous ulcers (more than 1 episode per year) and those who do not (No). No differences were observed between both study arms (p = 0.990).

**Table 2:** Percentage distribution of ABO and Rh blood group systems in our study versus in the general Lebanese population. Note the close agreement between the general blood group distribution in our study and the Lebanese population, suggesting that our sample is relatively representative of population’s blood group distribution.

Blood group (ABO Rh)	Total sample blood groups (n = 249) (percentage)	Individuals with recurrent ulcers in our sample (n = 72) (percentage)	Blood group distribution in Lebanese population^a^ (percentage)
A-	3.7	2.8	4.02
A+	40.8	41.7	33.86
AB-	0.5	4.2	0.43
AB+	5.2	1.4	3.64
B-	1.6	12.5	1.81
B+	13.1	4.2	17.79
O-	3.7	31.9	5.36
O+	31.4	2.8	33.09
Total	100.0	98.6	100.0

a From Tarhini et al. (2018), “Prevalence of principal ABO and rhesus blood group systems over the entire Lebanese population”.

Although the etiopathogenesis and predisposing factors of the recurrent oral ulcers, especially recurrent aphthous stomatitis, remain relatively unclear, it is, however, considered to be multifactorial, with major auto-inflammatory mediation.^8^

In our study, we attempted to assess different risk factors reportedly involved in recurrent aphthous ulcers, mainly ABO/Rh blood group systems, and evaluate the prevalence, age, and gender distribution of recurrent oral ulcers in a general Lebanese sample.

Our results show the prevalence of recurrent oral ulcers was (28.9%). Other studies reported similar prevalence results based on history.

In our study, the most frequently affected age group was 18‒25 years. These results do not contradict previous studies, which showed that RAS has a higher prevalence in younger adults, decreasing in both incidence and severity with age.^9^

Our study showed a slight gender predilection toward females (59.7%); other studies showed a higher prevalence of RAS in females as well.^9^

Stress appears to be a triggering factor for the onset of RAS; our results do not contradict this possible factor as 35% answered positively when they were asked if ulcers correlate with periods of stress (Fig. 2), and 23% were not sure.

**Figure 2:**
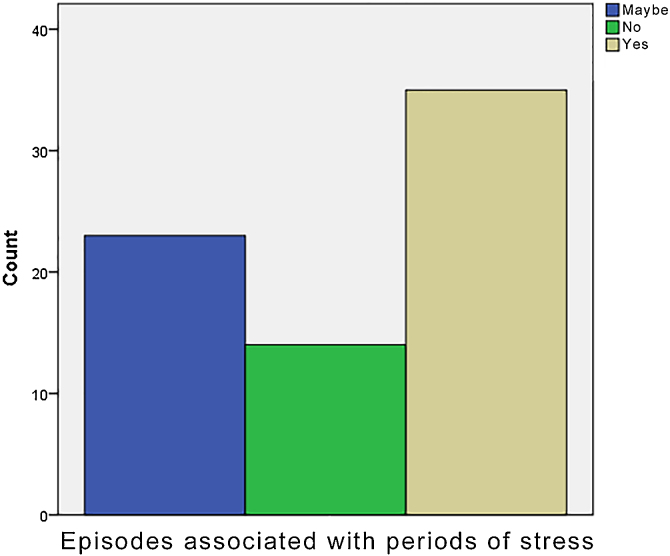
Episodes of oral ulcers coinciding with periods of stress.

Regarding nutritional factors, 31.9% of our participants have reported one or more hematinic deficiencies, while 19.4% did not reject nor ascertain.

In our study, 47.2% of patients suffering from RAS reported a positive familial history of the condition, while 15.3% were not sure. Similarly, in the study conducted by Abdullah, 34.4% of patients reported that another family member had suffered from RAU before.^10^

ABO/Rh blood group systems were studied in a few instances for potential association with the occurrence of recurrent aphthous ulcers. Association between the O blood group and peptic ulcers was the first evidence of this correlation. Our study showed no significant difference between patients with RAS among different ABO blood groups. Similar results were found in the study conducted by Sagiroglu et al. in 2018; although, they found that the risk of RAS was six times higher in B Rh(+) patients compared to B Rh(-) patients and three times higher in AB Rh(+) patients compared to AB Rh(-) patients; Similarly, other studies failed to find any statistically significant correlation between ABO blood groups and RAS.^1^, ^3^, ^6^, ^7^

To our knowledge, this is the first study to assess the prevalence of recurrent oral ulcers in the Lebanese population; moreover, it is one of the few conducted to assess the possible association of recurrent aphthous ulcers and ABO/Rh blood group systems. Nevertheless, the main limitations of our study include the self-reported nature of the questionnaire relying on participants’ histories and the relatively limited sample size; therefore, larger, prospective analytical and clinically correlated studies are warranted to confirm the results.

Recurrent oral ulcerations, with recurrent aphthous ulcers as the main culprit, remain one of the most common oral lesions associated with significant pain and discomfort for the patients. In our study, we were not able to prove an association between recurrent oral ulcers and ABO/Rh blood group systems; moreover, we reported epidemiological parameters of this entity in a Lebanese sample for the first time, which belongs to the reported global range.

## Financial support

None declared.

## Authors' contributions

Ziad Noujeim; Conception and methodology; planning of the study; significant collection of data; elaboration and writing of the manuscript; critical review of the literature; approval of final version.

Lara Nasr: Effective participation in research orientation; significant collection of data; elaboration and writing of the manuscript; critical review of the literature; approval of final version.

Racha Hajj: Conception and planning of the study; significant collection of data; elaboration and writing of the manuscript; critical review of the literature; approval of final version.

Abbass El-Outa: Conception and methodology; statistical analysis and interpretation of data; elaboration and writing of the manuscript; approval of final version.

## Conflicts of interest

None declared.
